# Sleep Problems Influence Emotional/Behavioral Symptoms and Repetitive Behavior in Preschool-Aged Children With Autism Spectrum Disorder in the Unique Social Context of China

**DOI:** 10.3389/fpsyt.2020.00273

**Published:** 2020-04-16

**Authors:** Yu-Qi Kang, Xiao-Rong Song, Geng-Fu Wang, Yuan-Yuan Su, Pei-Ying Li, Xin Zhang

**Affiliations:** ^1^ Department of Maternal, Child and Adolescent Health, School of Public Health, Tianjin Medical University, Tianjin, China; ^2^ Experimental Teaching Center of Preventive Medicine, Tianjin Medical University, Tianjin, China

**Keywords:** sleep disturbances, emotional/behavioral problems, repetitive behavior, autism spectrum disorder, Chinese preschool-aged children

## Abstract

Sleep disturbances are common in people with autism spectrum disorder (ASD), but research on this topic is still limited in China. In the current study, we evaluated the prevalence of sleep problems in preschool-aged children with ASD and to examine the correlations between sleep disturbances and emotional/behavioral symptoms and repetitive behavior in the unique social context of China. This study recruited 475 preschool-aged children aged 3–6 years old, including 252 children with ASD (mean age 5.13 ± 1.15, 80.6% male) and 223 age-matched typically developing (TD) children (mean age 5.12 ± 0.97, 74.9% male). The parents of all children completed a sociodemographic questionnaire and the Childhood Sleep Habits Questionnaire. The parents of 114 ASD children completed the Strengths and Difficulties Questionnaire (SDQ) and the Repetitive Behavioral Questionnaire-2 (RBQ-2). The prevalence of sleep problems in preschool-aged children with ASD in this study was 81.7%, which was higher than that in TD children (61.0%). The scores for bedtime resistance, sleep anxiety, sleep duration, parasomnias, and sleep onset delay in the ASD group were significantly higher than those in the TD group (t=−7.664, *P*=0.000; t=−10.477, *P*=0.000; t=−4.133, *P*=0.000; Z=−3.916, *P*=0.000; Z=−7.093, *P*=0.000; respectively). Sleep onset delay explained 17.3% of the variance (adjusted R^2^ = 0.173) in the total SDQ score of children with ASD, and bedtime resistance explained a large proportion of total RBQ-2 score variance (adjusted R^2^ = 0.206). The high rate of sleep disturbances in preschool-aged children with ASD emphasizes the importance of screening for sleep problems in this population. Attention should also be directed toward formulating good sleep hygiene practices for preschool-aged children in the particular social context and cultural setting of China.

## Introduction

Autism spectrum disorder (ASD) is a neurodevelopmental condition that has increased in prevalence over the past few decades, with a current rate of 1 in 59 children in the US (1). In China, the prevalence of ASD increased from 2.38/10,000 to 35.1/10,000 from 2006 to 2015 (2). ASD is characterized by impairments in two core domains: 1) social interaction and communication and 2) restricted and repetitive patterns of behavior, interests, and activities (3). ASD is often associated with comorbid conditions including emotional and behavioral dysregulation and psychiatric and medical issues [e.g., sleep disorders (4, 5)]. Previous studies have shown that the nature and severity of these impairments vary considerably among individuals on the spectrum; some experience relatively few of these problems, while many others exhibit one or more problems (6, 7). Being able to evaluate which problems are more likely to cause clinically significant impairments is particularly important. For this reason, researchers are increasingly interested in studying sleep disturbances related to clinically elevated impairments in children with ASD (8, 9).

The prevalence of sleep disturbances in children with ASD is as high as 50–80% in Western countries, which is approximately two or three times higher than the prevalence in typically developing (TD) children (20–50%) (4, 5, 10). An estimated 70–90% of Chinese children with ASD experience sleep disturbances (11, 12) compared to 15–80% of TD children (13, 14). Sleep problems were also reported more frequently among preschool-aged children with ASD compared to their TD peers in Western countries. Reynolds et al. found that 47% of parents in the US reported sleep problems in their children with ASD, compared with 25% in TD children (15). Moreover, sleep problems in TD children decreased with age but were more likely to persist in children with ASD (16, 17). These studies indicated that sleep patterns in children with ASD may change over time. Specifically, parents of younger children reported more problems with bedtime resistance, sleep anxiety, night wakings, and parasomnias, whereas parents of adolescents reported issues regarding sleep duration, daytime sleepiness, and sleep onset (16, 17). However, research on sleep problems in Chinese preschool-aged children is limited. Although one small study found no differences in the rates of sleep disturbances between Chinese preschool-aged children with ASD and their TD counterparts, parents of preschoolers with ASD reported more problems with sleep duration, struggles after night waking, and daytime fatigue than parents of school-aged children with ASD (18). A detailed examination of the characteristics of sleep problems in Chinese preschool-aged children with ASD may provide guidance for appropriate intervention strategies.

Sleep problems in children with ASD might be the result of interactions between biological, psychological, social/environmental, and familial factors (4). Certain comorbidities such as attention deficit hyperactivity disorder (ADHD) and anxiety are also risk factors for sleep disturbances (9). Biological and genetic abnormalities that might contribute to sleep problems in ASD include abnormalities in the hypothalamic-pituitary-adrenal axis regulating circadian rhythms and alterations in hormone/neurotransmitter (melatonin/serotonin) production (19–21). Previous studies have shown differences in the prevalence rates of sleep disturbances and sleep patterns across countries and cultures. Children with ASD and TD children from Eastern countries such as India and China seem to have higher rates of sleep disturbances than children in Western countries (11, 22, 23). This could be attributable to differences in sleeping habits between Western and Eastern countries; for example, cosleeping among children is very common in many Asian countries (14, 24). This practice has been associated with increased sleep difficulties (24) and is reportedly more frequent among children with ASD than among TD children (22).

Numerous studies have shown that a wide variety of persistent sleep difficulties experienced by ASD children exacerbate their core autistic symptoms (25) and are associated with many conditions that co-occur with ASD including increased anxiety, sensory over-responsivity (26), self-stimulatory behavior, and daytime challenging behavior (27), as well as medical comorbidities such as gastrointestinal problems (12) and epilepsy (28). Among children with ASD, poor sleepers exhibit more ritualistic, compulsive, irritable, and hyperactive behavior than good sleepers (29, 30). Sensory hypersensitivity in nighttime sleeping environments among children with ASD is associated with communication delays and an increased frequency of atypical behaviors (29). However, little is known about the association between sleep problems and emotional/behavioral problems or repetitive behavior in Chinese preschool-aged children with ASD.

The aim of the current study was to evaluate and compare sleep disturbances in preschool-aged children with ASD and TD children in the unique social context of China. We also examined the relationship between sleep disturbances and emotional/behavioral problems, as well as repetitive behavior.

## Material and Methods

### Participants

This cross-sectional study recruited participants over a 4-year period (2015–2018) in Tianjin in northern China. Children aged 3–6 years were diagnosed with ASD by experienced child psychiatrists specializing in this field using the Diagnostic and Statistical Manual of Mental Disorders Fifth Edition (DSM-5) criteria (4). The psychiatrists determined the severity of autism using the Childhood Autism Rating Scale (CARS). A total of 252 children with CARS scores ≥30 or higher were recruited for this study (31). A total of 223 age-matched TD children was recruited from kindergartens as control subjects. Medical histories were collected from the parents of children in both groups by trained researchers. Subjects were excluded if they had a diagnosis of chronic illness (gastrointestinal disease, chronic respiratory illnesses, epilepsy), fragile X syndrome, Down syndrome, tuberous sclerosis, cerebral palsy, and any mental disorders diagnosed by DSM-5 (e.g., ADHD, anxiety) or had used any medications that influence sleep, emotion, or behavior (e.g., methylphenidate, atypical antipsychotics, benzodiazepines) during the previous 6 months.

All participants' parents completed the Children's Sleep Habits Questionnaire (CSHQ) and a sociodemographic questionnaire. Although all 252 children with ASD were assessed with the Peabody Picture Vocabulary Test-Chinese edition (PPVT-C), only 114 successfully completed the test. The parents of these 114 participants completed the Strengths and Difficulties Questionnaire (SDQ) and the Repetitive Behavior Questionnaire-2 (RBQ-2), and these participants formed the ASD subgroup. Before the start of the study, we ensured that the researchers clearly and consistently understood the aims, methods, investigation procedures, and every item in the questionnaire through professional training. The parents of all the children were invited to complete the CSHQ at home within a 1-week window, and then we collected the questionnaires. Any confusion from the parents regarding the questionnaire was resolved promptly by members of the research team. After each questionnaire was completed, the research assistants immediately checked the data to ensure that no items were missing and that the answers to the questions were logical. The PPVT-C, SDQ, and RBQ-2 were completed at our assessment center.

Written informed consent was obtained from all the parents or caregivers who completed the questionnaires. This study was approved by the ethics committee of Tianjin Medical University.

### Materials

#### Sociodemographic Questionnaire

The parents were interviewed regarding their children's medical condition by trained researchers. The characteristics of the children (age, gender), and the families (parents' education level, parental age, family income) were assessed by sociodemographic questionnaire.

#### Children's Sleep Habits Questionnaire (CSHQ)

The CSHQ, a parent-reported measure widely used to evaluate recent sleep behavior in children (32), has been translated into Chinese and is extensively used to assess Chinese children (11, 24). The CSHQ is a 33-item questionnaire that provides a total score as well as eight subscale scores: Bedtime Resistance, Sleep Duration, Night Wakings, Sleep Onset Delay, Sleep Anxiety, Parasomnias, Sleep Disordered Breathing, and Daytime Sleepiness. All but eight items are rated on a three-point Likert scale (1= *rarely* for 0–1 time per week, 2=*sometimes* for 2–4 times per week, 3= *usually* if behavior occurs 5–7 times per week). The other eight items are measured with a three-point Likert scale: 1= *usually* or *not sleepy*, 2= *sometimes* or *very sleepy*, 3= *rarely* or *falls asleep*. Items that describe good sleep behaviors are reverse-scored. CSHQ scores >41 have been reported as the most sensitive cutoff for identifying sleep problems that warrant further clinical assessment. Cronbach's alpha coefficient for the CSHQ full scale was 0.86; for the subscales, it ranged from 0.38 (sleep disordered breathing) to 0.70 (sleep duration), with a median of 0.64 in a prior Chinese study (11).

#### Strengths and Difficulties Questionnaire (SDQ)

The 25-item SDQ was designed to measure emotional/behavioral problems (33). It has four difficulties or problems subscales (Emotional Symptoms, Conduct Problems, Hyperactivity, Peer Problems, and Prosocial Behavior) and one strengths subscale (Prosocial Behavior). Parents rate each item as 0, 1, or 2 for *not true*, *somewhat true*, and *definitely true*, respectively. The total difficulties score is acquired by summing the four problems subscale scores. Higher scores indicate more serious problems, except in the case of the Prosocial Behavior subscale. The Chinese version of this questionnaire has been shown to have good reliability and validity (11, 24, 34). In the current study, Cronbach's alpha coefficient for the SDQ total difficulties scale was 0.641.

#### Repetitive Behavioral Questionnaire-2 (RBQ-2)

The RBQ-2 is suitable for 2- to 8-year-old children and includes 20 items (35). A large number of children with ASD were used to evaluate repetitive behaviors. Parents rated each item from 1 to 3. The questionnaire has been divided into different subscales for different studies (using a 2-, 3-, and 4-factor structure). We used four subscales (Repetitive Motor Movements, Rigidity, Preoccupation with Restricted Patterns of Interest, and Unusual Sensory Interest) to provide the best possible explanation. The RBQ-2 has good internal consistency and validity. In the current study, Cronbach's alpha coefficient for the RBQ-2 full scale was 0.717.

#### Childhood Autism Rating Scale (CARS)

The CARS has been widely used to assess the severity of autism and is administered by experienced pediatric psychiatrists (31). It consists of 15 items, and each item is scored from 1 to 4. The CARS measures behaviors such as relating to people, imitation, adaptation to change, verbal and nonverbal communication, sensory sensitivities, body and object use, activity, IQ level, and general impressions. The total score range is between 15 and 60, with higher scores indicating more severe levels of autism. It has been validated in studies in Chinese children with ASD (2, 12, 36). The Cronbach's alpha coefficients for the CARS were 0.735 in a prior Chinese study (37) and 0.83 in our study.

#### Peabody Picture Vocabulary Test-Chinese Edition (PPVT-C)

The PPVT-C is a commonly used test that evaluates receptive verbal ability (38) as means of assessing children's intellectual development (39). The test shows a set of four pictures at a time, and the examinee is asked to select the correct picture. The PPVT-C has shown reliability and validity in recent studies in China (12, 39), and in our previous study, we also used the PPVT-C to evaluate cognitive ability in children with ASD (36).

### Data Analysis

All analyses were conducted using SPSS 24.0 (IBM Corporation, Armonk, NY, USA). Descriptive statistics were presented using the mean and standard deviation, median and maximum, and minimum for continuous variables, and frequencies and percentages for categorical variables. The ASD and TD groups were categorized based on the total CSHQ score: those with >41 were classified as poor sleepers, and those with ≤41 were classified as good sleepers. Group differences in demographic data, participant characteristics, and sleep characteristics were analyzed using chi-square tests for categorical variables and independent-sample t-tests and Mann-Whitney U tests for continuous variables. Analyses of variance (ANOVAs) were performed to compare the CSHQ scores for each age group (3, 4, 5, and 6 years) within the ASD and TD groups.

In the ASD subgroup that completed the PPVT-C, the SDQ, and the RBQ, Pearson's correlations for normal continuous variables and Spearman's correlations for nonnormally distributed variables were calculated to detect correlations among the CSHQ subscales, the SDQ subscales, and the RBQ-2 subscales. To test whether the CSHQ subscale scores and total score explain the total SDQ and RBQ-2 scores in the ASD subgroup, 18 regression models were generated after adjusting for age, gender, family income, CARS score, and PPVT-C score. We used 18 linear regression analyses with the total SDQ and RBQ-2 scores as the dependent variables and the scores from each CSHQ subscale (i.e., bedtime resistance, sleep anxiety, sleep duration, sleep disordered breathing, parasomnias, daytime sleepiness, night wakings, and sleep onset delay) and the total CSHQ score as independent variables. Finally, two multiple linear regression models with the total SDQ and RBQ-2 scores as the dependent variables included all the CSHQ subscale scores. Statistical significance was set at *P* < 0.05.

## Results

### Sociodemographic Characteristics

In the ASD group, the mean age was 5.13 ± 1.15 years, and 80.6% were male. In the TD group, the mean age was 5.12 ± 0.97 years, and 74.9% were male. There were no statistically significant differences between the ASD and TD groups for gender, age, parental age, parents' education level, or family income (**Table 1**).

**Table 1:** Sociodemographic characteristics of participants.

	ASD(n=252) Mean ± SD/n(%)	TD(n=223) Mean ± SD/n(%)	t*/χ* ^2^	*P*
Age(years)	5.13 ± 1.15	5.12 ± 0.97	-0.174	0.862
**Gender**			2.207	0.137
Male	203(80.6)	167(74.9)		
Female	49(19.4)	56(25.1)		
Paternal age	34.78 ± 4.55	34.58 ± 4.86	-0.461	0.645
Maternal age	33.58 ± 3.90	33.06 ± 4.62	-1.322	0.187
**Paternal education level**			0.030	0.863
High school or below	101(40.2)	88(39.5)		
Undergraduate or above	150(59.8)	135(60.5)		
**Maternal education level**			0.303	0.582
High school or below	84(33.5)	80(35.9)		
Undergraduate or above	167(66.5)	143(64.1)		
**Monthly family income**			1.797	0.407
< 3000 RMB*	30(12.0)	20(9.0)		
3000–6,000 RMB*	91(36.3)	87(39.0)		
> 6,000 RMB*	130(51.8)	116(52.0)		

*1 RMB≈0.14 US Dollars

ASD, autism spectrum disorder; TD, typically developing.

### Sleep Disturbances in Children With ASD

**Table 2** shows the CSHQ scores and the number above the cutoff. Children with ASD exhibited a significantly higher mean total score on the CSHQ (47.15 ± 5.39; t=−7.905, df=473, *P*=0.000). The skewness and kurtosis values of sleep disordered breathing, parasomnias, night wakings, and sleep onset delay in the TD group were 2.44 and 5.11, 1.48 and 1.38, 2.72 and 6.72, and 1.55 and 1.35, respectively, and the corresponding values of these four subscales in the ASD group were 2.51 and 6.56, 1.40 and 1.54, 2.74 and 8.51, and 0.34 and −1.24, respectively. All other skewness and kurtosis values ranged from −1 to 1 and were close to the normal distribution. Independent-samples t-tests for normal continuous variables and Mann-Whitney U tests for nonnormally distributed variables were performed to compare CSHQ subscale scores between the ASD and TD groups. Bonferroni-adjusted alpha levels (*P* < 0.0056) were calculated in independent-sample t-tests and Mann-Whitney U tests for *post hoc* pairwise comparisons. After correction, we found that the subscale scores for bedtime resistance, sleep anxiety, sleep duration, parasomnias, and sleep onset delay in the ASD group were still significantly higher than those in the TD group.

**Table 2:** Sleep problems in the ASD group compared to the TD group.

CSHQ domains	ASD (n=252)		TD (n=223)		*P* ^a^	*P* ^b^
	Mean ± SD/Median(min-max)	n(%)	Mean ± SD/Median(min-max)	n(%)		
Bedtime resistance	11.67 ± 2.17	229(90.9)	9.85 ± 2.89	140(62.8)	**0.000**	0.000
Sleep anxiety	6.95 ± 1.77	231(91.7)	5.34 ± 1.56	114(51.1)	**0.000**	0.000
Sleep duration	4.49 ± 1.62	145(57.5)	3.96 ± 1.15	111(49.8)	**0.000**	0.090
Sleep disordered breathing	3(3–5)	50(19.8)	3(3–6)	36(7.6)	0.308	0.296
Parasomnias	8(7–13)	148(58.7)	7(7–12)	86(38.6)	**0.000**	0.000
Daytime sleepiness	11.77 ± 2.68	153(60.7)	11.35 ± 2.43	133(59.6)	0.073	0.812
Night wakings	3(3–9)	64(25.4)	3(3–6)	39(17.5)	0.110	0.037
Sleep onset delay	2(1–3)	149(59.1)	1(1–3)	62(27.8)	**0.000**	0.000
Total score	47.15 ± 5.39	206(81.7)	43.05 ± 5.92	136(61.0)	**0.000**	0.000

^a^Mean score/median score comparisons between the ASD group and the TD group.

^b^Rate comparisons between the ASD group and the TD group.

Bolded P values were significant after Bonferroni correction (P＜0.0056).

ASD, autism spectrum disorder; TD, typically developing.

A total CSHQ >41 is considered a sensitive cutoff when screening children for sleep problems. There were 206 (81.7%) children with ASD who had scores above this cutoff, which was significantly different from the TD group (61.0%; *χ* ^2^ = 25.290, df=1, *P*=0.000). Furthermore, the proportions of subscale scores above the cutoff for bedtime resistance, sleep anxiety, parasomnias, and sleep onset delay were significantly higher in the ASD group than in the TD group (*χ* ^2^ = 53.859, df=1, *P*=0.000; *χ* ^2^ = 97.842, df=1, *P*=0.000; *χ* ^2^ = 19.247, df=1, *P*=0.000; *χ* ^2^ = 47.019, df=1, *P*=0.000; respectively) (**Table 2**).

ANOVAs were used to compare the total CSHQ scores for each age group (3, 4, 5, and 6 years); there were no significant differences among age groups within the ASD (F=0.536, df=3, *P*=0.658) or TD groups (F=0.901, df=3, *P*=0.441). The difference in family income between good sleepers (total CSHQ score >41) and poor sleepers (≤41) in the ASD group was statistically significant (*χ* ^2^ = 7.013, df=2, *P*=0.030) (**Table 3**).

**Table 3:** Sociodemographic characteristics of good sleepers compared to poor sleepers.

	ASD(n = 252)				TD(n = 223)		t*/χ* ^2^	*P*
	Good sleepers n = 46 Mean ± SD/n(%)	Poor sleepers n = 206 Mean ± SD/n(%)	t*/χ* ^2^	*P*	Good sleepers n = 87 Mean ± SD/n(%)	Poor sleepers n = 136 Mean ± SD/n(%)	t*/χ* ^2^	*P*
Age(years)	5.19 ± 1.02	5.12 ± 1.18	0.381	0.704	5.21 ± 0.92	5.06 ± 0.10	1.168	0.244
**Gender**			0.189	0.664			0.342	0.559
Male	36(78.3)	167(81.1)			67(77.0)	167(74.9)		
Female	10(21.7)	39(18.9)			20(23.0)	56(25.1)		
Paternal age	35.17 ± 6.22	34.69 ± 4.10	0.646	0.519	34.53 ± 4.76	34.62 ± 4.93	−0.133	0.894
Maternal age	33.37 ± 3.73	33.62 ± 3.94	−0.395	0.693	33.24 ± 5.33	32.94 ± 4.12	0.472	0.637
**Paternal education level**			1.262	0.261			0.562	0.454
High school or below	22(47.8)	80(38.8)			50(57.5)	51(37.5)		
Undergraduate or above	24(52.2)	126(61.2)			37(42.5)	85(62.5)		
**Maternal education level**			3.578	0.059			0.637	0.425
High school or below	21(45.7)	64(31.1)			34(39.1)	46(33.8)		
Undergraduate or above	25(54.3)	142(68.9)			53(60.9)	90(66.2)		
**Monthly family income**			7.013	0.030			2.467	0.291
< 3,000 RMB*	8(17.4)	22(10.7)			10(11.5)	10(7.4)		
3,000–6,000 RMB*	9(19.6)	82(39.8)			3742.5)	50(36.8)		
> 6,000 RMB*	29(63.0)	102(49.5)			40(46.0)	76(55.9)		

*1 RMB≈0.14 US Dollars.

ASD, autism spectrum disorder; TD, typically developing.

### Description of the Findings for Each CSHQ Item in Children With ASD

**Table 4** shows the percentage of “usually” responses for each item on the CSHQ to fully characterize the differences in sleep characteristics between children with ASD and TD children. On the bedtime resistance subscale, the ASD group exhibited significantly more frequent sleep problems than the TD group for all items except “struggles at bedtime” (*χ* ^2^ = 0.086, *P*=0.769). On the sleep anxiety subscale, only “has problems sleeping outside the home” (*χ* ^2^ = 0.790, *P*=0.347) was not significantly different between the ASD and TD groups. On the sleep duration subscale, children with ASD exhibited a significantly lower score for “sleeps a moderate amount” (*χ* ^2^ = 36.272, *P*=0.000) compared with TD children. On the parasomnias subscale, children with ASD more frequently exhibited the characteristics “wets bed at night” (*χ* ^2^ = 6.888, *P*=0.009) and “restless during sleep” (*χ* ^2^ = 7.744, *P*=0.005) than TD children. On the sleep onset delay subscale, children with ASD exhibited a lower score for “falls asleep in 20 minutes” (*χ* ^2^ = 47.019, *P*=0.000) than TD children. Although the daytime sleepiness and night wakings subscales were not significantly different between groups, children with ASD exhibited a significantly higher rate of “sleepy/has fallen asleep while watching TV” and “awakes once at night” than TD children.

**Table 4:** CSHQ subscale items for children with ASD and TD children.

Each items and subscales of the CSHQ	Response of “usually” n (%)		*χ* ^2^	*P*
	ASD (n = 252)	TD (n = 223)		
**Bedtime resistance**				
Goes to bed at same time at night	178(70.6)	181(81.2)	7.109	0.008
Falls asleep in own bed	36(14.3)	101(45.3)	55.417	0.000
Sharing bed with parent/sibling	94(37.3)	57(25.6)	7.521	0.006
Needs person in room to fallasleep	188(74.6)	39(17.5)	154.672	0.000
Struggles at bedtime	11(4.4)	11(4.9)	0.086	0.769
Afraid of sleeping alone	89(35.3)	28(12.6)	33.016	0.000
**Sleep anxiety**				
Fears of sleeping in darkness	35(13.9)	13(5.8)	8.459	0.004
Has problems for sleeping outside the home	3(1.2)	5(2.2)	0.790	0.374
**Sleep duration**				
Decreased sleep duration	22(8.7)	13(5.8)	1.458	0.227
Sleeps in the moderate amount	135(53.6)	178(79.8)	36.272	0.000
Sleeps in the same amount each day	170(67.5)	165(74.0)	2.427	0.119
**Sleep disordered breathing**				
Snores loudly	7(2.8)	8(3.6)	0.254	0.615
Stops breathing during sleep	0	0	–	–
Snorts and gasps during sleep	5(2.0)	2(0.9)	0.963	0.326
**Parasomnias**				
Wets bed at night	15(6.0)	3(1.3)	6.888	0.009
Talks during sleep	3(1.2)	2(0.9)	0.098	0.754
Restless during sleep	29(11.5)	10(4.5)	7.744	0.005
Sleepwalks at night	1(0.4)	0	0.887	0.346
Grinds teeth during sleep	13(5.2)	5(2.2)	2.760	0.097
Awakes with creaming, sweating	3(1.2)	5(2.2)	0.790	0.374
Awakes with nightmare	1(0.4)	4(1.8)	2.216	0.137
**Daytime sleepiness**				
Wakes up by him/herself	106(42.1)	95(42.6)	0.014	0.906
Wakes up in negative mood	9(3.6)	6(2.7)	0.300	0.584
Wakes up by another person	44(17.5)	47(21.1)	0.999	0.318
Difficulty getting out of bed	16(6.3)	8(3.6)	1.881	0.170
Takes a long time to be alert in morning	9(3.6)	5(2.2)	0.731	0.393
Seems tired	4(1.6)	3(1.3)	0.048	0.827
Sleepy/falls asleep while watching TV	39(15.5)	17(7.6)	7.015	0.008
Sleepy/falls asleep while riding car	101(40.1)	80(35.9)	0.887	0.346
**Night wakings**				
Moves to parents'/sibling's bed at night	7(2.8)	2(0.9)	2.252	0.133
Awakes once at night	16(6.3)	3(1.3)	7.714	0.005
Awakes more than once at night	10(4.0)	4(1.8)	1.956	0.162
**Sleep onset delay**				
Falls asleep within 20 minutes	103(40.9)	161(72.2)	47.019	0.000

Two items that are duplicated in the anxiety subscale are not repeated here.

CSHQ, Children's Sleep Habits Questionnaire; ASD, autism spectrum disorder; TD, typically developing.

### Relationship Between Sleep Disturbance and Daytime Functions in the ASD Subgroup

The participants with ASD who successfully completed the PPVT-C formed the ASD subgroup. In-depth analyses (**Table S1**) showed that children with ASD who completed the PPVT-C were older (t=3.197, *P*=0.002) and had less severe autism than those who did not complete the PPVT-C (t=−3.863, *P*=0.000). Although there was no significant difference in family income between these groups, there was a trend in which ASD patients from higher-income families were more likely to complete the test (χ^2^ = 5.935, *P*=0.051).

In the ASD subgroup, Pearson's correlations for normal continuous variables and Spearman's correlations for nonnormally distributed variables were calculated to detect correlations among the CSHQ, SDQ, and RBQ-2 domains in the ASD subgroup. The skewness and kurtosis values for sleep disordered breathing, parasomnias, night wakings, sleep onset delay, conduct problems, repetitive movements, and rigidity were 2.70 and 6.52, 1.25 and 0.93, 2.48 and 6.87, 0.60 and −1.35, 1.04 and 1.53, 1.58 and 2.77, and 1.10 and 1.91, respectively. All other skewness and kurtosis values ranged from −1 to 1 and were close to the normal distribution.

The correlations between each SDQ domain and all CSHQ domains in the children with ASD are shown in **Table 5**. Some significant but relatively weak positive correlations (*P* < 0.05) were found, including between the total SDQ score and bedtime resistance, parasomnias, and night wakings. Some significant moderate correlations (*P* < 0.01) were observed, such as between the total SDQ score and sleep anxiety. In addition, some highly significant correlations (*P* < 0.001) were found, such as those between the total SDQ score and sleep onset delay and the total CSHQ score.

**Table 5:** Relationships between the CSHQ and the SDQ, RBQ-2 domains, CARS, and PPVT-C.

	CSHQ subscale								
	Bedtime resistance	Sleep anxiety	Sleep duration	Sleep disordered breathing	Parasomnias	Daytime sleepiness	Night wakings	Sleep onset delay	Total score
Emotional symptoms	0.181	0.164	0.223*	0.002	0.191*	0.330***	0.128	0.240*	0.408***
Conduct problems	0.219*	0.104	0.175	−0.152	−0.026	0.054	0.123	0.221*	0.166
Hyperactivity	0.241*	0.425***	−0.061	0.127	0.235*	0.083	0.126	0.201*	0.247**
Peer problems	0.066	0.098	−0.034	−0.037	0.055	−0.108	0.188*	0.159	0.033
Prosocial behavior	−0.020	−0.204*	0.037	−0.258**	−0.057	0.110	−0.127	−0.111	−0.060
Total SDQ score	0.196*	0.312**	0.126	0.033	0.185*	0.128	0.231*	0.350***	0.324***
Repetitive movements	0.193*	0.265**	0.105	0.237*	0.179	0.113	0.235*	0.185*	0.317**
Rigidity	0.614***	0.143	0.054	0.114	0.116	−0.076	0.079	0.182	0.291**
PRI	0.278**	0.171	0.000	0.229*	0.314**	0.166	0.009	0.143	0.334***
Sensory interest	0.315***	0.282**	−0.037	0.159	0.256**	0.172	0.098	−0.007	0.339***
Total RBQ-2 score	0.433***	0.289**	0.070	0.292**	0.244**	0.109	0.167	0.192*	0.431***
CARS	0.196*	0.412***	0.221*	0.211*	0.167	0.185*	0.291**	0.321**	0.393***
PPVT-C	−0.279**	−0.486***	−0.085	−0.118	−0.276**	−0.134	−0.103	−0.187*	−0.349***

Pearson's or Spearman's correlation coefficients were computed for the ASD subgroup.

Spearman's correlations were computed for nonnormally distributed variables (sleep disordered breathing, parasomnias, night wakings and sleep onset delay, conduct problems, repetitive movements and rigidity).

*P < 0.05, **P < 0.01, ***P < 0.001.

CSHQ, Children's Sleep Habits Questionnaire; SDQ, Strengths and Difficulties Questionnaire; PRI, preoccupation with restricted patterns of interests; RBQ-2, Repetitive Behavioral Questionnaire-2; CARS, Childhood Autism Rating Scale; PPVT-C, Peabody Picture Vocabulary Test-Chinese edition.

We generated nine linear single-predictor regression models that included the total scores for each CSHQ domain and adjusted for gender, age, family income, PPVT-C score, and CARS score (**Table 6**). **Table 6** shows that sleep onset delay scores had the greatest ability to explain the total SDQ score in children with ASD (adjusted R^2^ = 0.173). Finally, we performed a multiple linear regression analysis that included scores for the eight CSHQ subscales as independent variables. The incorporation of the total scores from these subscales into a single regression model yielded an adjusted R^2^ = 0.207 (**Table 7**). After Bonferroni correction (*P*=0.05/8 sleep problems), sleep onset delay was also significant (*P*=0.003).

**Table 6:** SDQ and RBQ-2 total scores by each domain of the CSHQ: adjusted linear regression results.

	SDQ total score				RBQ-2 total score				
	Adj.R^2^	β	95% CI	*P*	Adj.R^2^	β	95% CI	*P*	VIF
Bedtime resistance	0.123	0.133	(−0.133-0.821)	0.155	0.206	0.402	(0.464-1.189)	0.000	1.121
Sleep anxiety	0.140	0.214	(0.024-1.288)	0.042	0.088	0.213	(0.001-1.041)	0.049	1.426
Sleep duration	0.109	0.048	(−0.418-0.721)	0.599	0.054	0.014	(−0.433-0.503)	0.882	1.055
Sleep disordered breathing	0.108	-0.042	(−2.587-1.614)	0.647	0.152	0.314	(1.263-4.534)	0.001	1.062
Parasomnias	0.143	0.196	(−0.056-1.521)	0.035	0.169	0.347	(0.540-1.692)	0.000	1.111
Daytime sleepiness	0.112	0.076	(−0.223-0.551)	0.403	0.060	0.074	(−0.190-0.446)	0.426	1.041
Night wakings	0.107	0.023	(−0.843-1.069)	0.815	0.060	0.081	(−0.460-1.106)	0.415	1.185
Sleep onset delay	0.173	0.266	(0.581-2.978)	0.004	0.069	0.127	(−0.336-1.694)	0.188	1.119
Total CSHQ score	0.151	0.231	(0.036-0.400)	0.019	0.189	0.401	(0.160-0.444)	0.025	1.256

Adjusted for age, gender, family income, CARS score, and PPVT-C score.

SDQ, Strengths and Difficulties Questionnaire; RBQ-2, Repetitive Behavioral Questionnaire-2; Adj.R^2^, adjusted R^2^; CSHQ, Children's Sleep Habits Questionnaire.

**Table 7:** Multiple linear regression analysis of CSHQ subscales as independent variables.

**Independent variable**	Dependent variable: total SDQ score			Dependent variable: total RBQ-2 score			
	β	95% CI	*P*	β	95% CI	*P*	VIF
Bedtime resistance	0.060	(−0.327-0.636)	0.525	0.323	(0.304-1.024)	**0.000**	1.251
Sleep anxiety	0.181	(−0.108-1.214)	0.100	0.141	(−0.149-0.839)	0.169	1.583
Sleep duration	−0.079	(−0.874-0.379)	0.435	−0.100	(−0.721-0.216)	0.288	1.461
Sleep disordered breathing	−0.138	(−3.788-0.594)	0.151	0.206	(0.265-3.542)	0.023	1.140
Parasomnias	0.201	(0.005-1.612)	0.049	0.194	(0.021-1.223)	0.043	1.313
Daytime sleepiness	0.005	(−0.370-0.391)	0.956	0.016	(−0.257-0.312)	0.849	1.213
Night wakings	0.058	(−0.682-1.261)	0.556	0.158	(−0.097-1.356)	0.089	1.251
Sleep onset delay	0.299	(0.687-3.306)	**0.003**	0.095	(−0.474-1.485)	0.309	1.577
Adjusted R^2^	0.207			0.304			
*P*	0.014			0.000			

The children's age, gender, family income, CARS score, and PPVT-C score were entered as control variables in the first step; adjusted R^2^ = 0.115, P = 0.003.

All CSHQ subscale scores are included in the model.

Bolded P values were significant after Bonferroni correction (P < 0.0063).

SDQ, Strengths and Difficulties Questionnaire; RBQ-2, Repetitive Behavioral Questionnaire-2.

**Table 5** also shows the correlations between each RBQ-2 domain and all CSHQ domains in the ASD group. Some significant but relatively weak positive correlations (*P* < 0.05) were found, including between the total RBQ-2 score and sleep onset delay. We also observed significant moderate correlations (*P* < 0.01), such as the associations between the total RBQ-2 score and sleep anxiety, sleep disordered breathing, and parasomnias. In addition, highly significant correlations (*P* < 0.001) were revealed, such as the associations of the total RBQ-2 score with bedtime resistance and the total CSHQ score.

Nine additional regression models were constructed to examine how much variance was explained by the CSHQ; these models included the total RBQ score as the outcome variable. Notably, bedtime resistance explained a large proportion of variance in RBQ-2 scores (adjusted R^2^ = 0.206, **Table 6**). The incorporation of the total raw scores from all eight CSHQ subscales into a single regression model yielded an adjusted R^2^ value of 0.304 (**Table 7**). After Bonferroni correction, the *P* value of bedtime resistance was significant (*P*=0.000).

## Discussion

Sleep disturbances in preschool-aged children with ASD and their associations with daily behavior in children have been widely researched in the West; however, research on these topics is still lacking in China. This was the first cross-sectional study to compare sleep problems between TD children and Chinese preschool-aged children with ASD with sleep problems who were not taking any medications. We evaluated the prevalence and characteristics of sleep disturbances using the CSHQ and explored the relationship between sleep problems and emotional/behavioral problems, as well as repetitive behavior in children with ASD.

The prevalence rates of sleep disturbances in children with ASD and TD children were previously reported to 50–80% (4, 5, 10) and 20–50% (4, 32), respectively. The wide ranges may be attributed to differences in clinical criteria, the questionnaires used, population characteristics, and sample sizes. In the present study, we used the CSHQ cutoff of 41 and found that 81.7% of parents reported sleep problems in preschool-aged children with ASD, which was higher than the results for TD children (61.0%). Some studies have indicated that the CSHQ may overestimate sleep problems in young children (15). When we applied the more conservative CSHQ cutoff of 48 (15), 42.1% of preschool-aged children with ASD had sleep problems, compared with 17.5% of TD children. Using a cutoff of 48, our findings were consistent with a previous US study of 2- to 5-year-old children in which researchers found 47% of parents reported sleep problems in their child with ASD, and 25% in TD children (15). Regardless of the cutoff used, sleep problems are more common in preschool-aged children with ASD than in TD controls. Further studies are necessary to determine the optimal clinical cutoff for preschool-aged children in China, as such information will better validate the CSHQ as a screening tool for assessing sleep problems in this population.

In addition, over 90% of the preschool-aged children with ASD scored above the cutoffs on the CSHQ subscales of bedtime resistance and seep anxiety. Reduced sleep duration, parasomnias, daytime sleepiness, and sleep onset delay were reported in more than 50% of the preschool-aged children with ASD (**Table 2**). These findings are consistent with previous studies demonstrating that reduced sleep duration and difficulties in sleep onset and maintenance were the predominant concerns expressed by parents of preschool-aged children with ASD (16–18).

To explore sleep patterns in greater detail, we further analyzed differences between the ASD and TD groups on each CSHQ subscale and item. We found that differences in the characteristics of sleep disturbances in children with and without ASD were reflected by significant group differences on five of the eight CSHQ subscales (bedtime resistance, sleep anxiety, sleep duration, parasomnias, and sleep onset delay). Parent reports indicated that children with ASD presented higher levels of bedtime resistance than TD children. Item-level comparisons of the results for the CSHQ bedtime resistance subscale showed that children with ASD were more likely to share a room or bed with their parents. However, there were no differences in struggles at bedtime. These results suggest that the difference might be largely due to the higher rate of cosleeping among children with ASD. A cross-cultural study showed that the rates of room- and bed-sharing among Chinese preschool-aged children were 93.1% and 70.1%, respectively (40). Nevertheless, data on cosleeping for Chinese preschool-aged children with ASD are limited. In our study, parents more often responded “usually” for the items “needs a person in the room to fall asleep” and “does not fall asleep in own bed” for preschoolers with ASD. Cosleeping was considered the strongest predictor of parent-reported sleep problems and correlated with increased bedtime resistance, sleep anxiety, night wakings, and daytime sleepiness (41) in school-aged TD children. In the present study, sleep anxiety occurred more frequently in children with ASD, whereas there were no significant differences in night wakings or daytime sleepiness. Some studies found that cosleeping with parents increased the risk of sleep disturbances in children with ASD (22, 23), but Wang et al. reported that the practice of cosleeping was not associated with sleep disturbances in school-aged children with ASD (11). Because of intrinsic sociocultural values and socioeconomic factors in China, cosleeping is a normal practice (42), and further study is urgently needed to explore the inconsistent relationship between cosleeping and sleep disturbances in Chinese preschool-aged children with ASD.

We found that reduced sleep duration and sleep onset delay were more severe in preschool-aged children with ASD. On the CSHQ sleep duration subscale, children with ASD exhibited significantly lower rates of “usually” responses for “sleeps a moderate amount” compared with TD children. These differences may be due to the core symptoms of ASD, which hamper the establishment of appropriate sleep routines. For example, compulsions and rituals may influence bedtime resistance; children with ASD may ruminate, leading to delayed sleep onset (9); and impaired social communication may make it difficult for children with ASD to understand their parents' expectations regarding bedtime (43). These results may also be attributable to abnormal melatonin secretion, which is a common trait in ASD (21). This could make it difficult for children with ASD to establish sleep-wake rhythms (21). In addition, on the parasomnias subscale, parents of children with ASD were more likely to indicate that their child “usually” “wets the bed at night” and is “restless during sleep.” The high prevalence of restlessness might be because parents who share beds with the child are more likely to be aware of the child's restlessness at night (23).

Notably, we did not find a significant difference in the night wakings subscale between groups. This result was consistent with a Chinese study demonstrating that scores on the night wakings subscale did not differ significantly between preschool-aged children with ASD and TD children (18). However, Wang et al. reported that school-aged Chinese children with ASD were more likely to exhibit problems with night wakings (11). Interestingly, item-level comparisons of night wakings in our study showed that children with ASD were more likely to awake once at night. Some studies demonstrated that a lengthy period of night waking in children with ASD was more unusual than the frequency of waking (4, 18).

We found that sleep disturbances were not significantly associated with age in preschool-aged children with ASD. Wang et al. evaluated a sample of 60 children with ASD (aged 6–17 years) and reported findings that were consistent with our results (5). Some studies have found that total sleep problems are persistent in ASD (16, 17), whereas others concluded that age is a predictor of sleep problems (24, 44). We did not find gender-related differences in Chinese preschool-aged children with ASD, which is consistent with previous studies (45, 46). In contrast, Wang et al. found that school-aged girls were more likely than boys to have sleep disturbances (11). Our study also suggested that sleep disturbances in Chinese preschool-aged children with ASD are associated with family income. This is consistent with previous studies that found that children from families with a lower socioeconomic status (SES) experienced more sleep problems than those from families with a higher SES (45). In contrast, some studies failed to find significant SES differences associated with sleep disturbances (11, 46, 47). In light of these inconsistent findings, further research might examine the associations between sociodemographic factors and sleep disturbances in a large longitudinal study of Chinese children with ASD.

The correlation between sleep problems and cognitive function in children with ASD remains controversial. One study reported that sleep disturbances were associated with impaired cognitive ability (12), while another disagreed (44). In the current study, PPVT-C scores were significantly negatively correlated with the total CSHQ score in preschool-aged children with ASD. Further studies with larger samples should clarify these inconsistencies. There is also no agreement regarding a relationship between sleep disturbances and ASD severity. One group found that bedtime resistance and night wakings were positively correlated with the total CARS score (48). Another demonstrated that autism severity predicted increases in sleep problems (49). However, another report found no association between sleep disturbances and ASD severity (44). In our study, CARS scores were significantly positively correlated with all CSHQ domains except the parasomnias subscale. The inconclusiveness might be due to other factors that affect sleep and autistic symptom severity, such as medications or family factors (44). Therefore, we adjusted for gender, age, family income, and PPVT-C and CARS scores and attempted to isolate ASD from comorbid conditions that are likely to influence sleep problems (8, 9) to explore how and to what extent sleep problems influence emotional/behavioral problems and repetitive behavior in preschool-aged children with ASD.

The findings of the present study and a previous report suggest that sleep problems are associated with emotional/behavioral problems in children with ASD (11). Significant correlations were found between the total CSHQ score and emotional symptoms, hyperactivity, and the total SDQ score. Notably, sleep onset delay explained a considerable proportion (17.3%) of the variance in the total SDQ score of preschool-aged children with ASD. In a prior study of children with ASD who were classified by their parents as good or bad sleepers, the latter were reported to have hyperactivity, anxiety, and attention problems (17). Meanwhile, we found significant correlations between the total CSHQ score and the total RBQ-2 score and all its domains (e.g., repetitive movements, rigidity, preoccupation with restricted patterns of interests, and sensory interest). Linear regression showed that bedtime resistance explained a large proportion of the variance in the total RBQ-2 score (20.6%), indicating that bedtime resistance is a risk factor for repetitive behavior in preschool-aged children with ASD. These findings were consistent with prior findings that sleep disturbances influenced repetitive behavior (9, 45). The use of supplemental melatonin or behavioral interventions to treat insomnia might improve daytime impairment (50). Therefore, there might be a subtype of autism that co-occurs with sleep disturbances and is related to clinically elevated problem behavior and emotional/behavioral symptoms. Improved understanding of the association between specific sleep problem patterns and daytime function in children with ASD will provide guidance for tailoring medical care and behavioral sleep education for children with ASD.

Sleep disturbances are normal in preschool-aged children with ASD; however, symptoms of insomnia are often neglected by their parents. Many clinical doctors have limited knowledge of sleep problems (51), and the US Food and Drug Administration (FDA) did not approve sleep medications for children until 2017 (52). The Autism Treatment Network (ATN) sleep committee experts presented a consensus statement that all children with ASD should be screened for insomnia, that treatment should begin with the parents, and that behavioral interventions should be the first-line approach (50). Thus, China should screen for sleep problems in all children with ASD regardless of cognitive level and age. Additionally, we should encourage the establishment of a special sleep routine for preschool-aged children with ASD based on the Chinese social context.

There were some limitations in our study. First, we employed a cross-sectional design that relied on parental reports. It is not clear that the relationship between sleep disturbances and emotional/behavioral symptoms is causal. In addition, there is no evidence that the CSHQ is an appropriate instrument for use in children <4 years of age or those with ASD. Reynolds et al. suggested a higher CSHQ cutoff score for younger children (15). However, there is also modest evidence to suggest that holistic parent report measures such as the CSHQ are a superior single-item response measure that help gauge the overall sleep quality of children with ASD (53). Other researchers used the CSHQ to assess sleep in children <4 years and those with ASD (12, 16, 17). In the current study, the Cronbach's alpha coefficient for the full CSHQ scale was 0.72, indicating that it was reliable. Furthermore, although parental reports are usually dependable, parents in China often pay close attention to their children's sleep habits (24), and this could lead to overestimation of sleep problems. We also relied on subjective reports, which may increase the possibility of an association due to shared method variance. Therefore, it is necessary to interpret our findings with some caution. Future studies that employ objective measures of sleep would therefore be useful. Additionally, in our study, not all ASD diagnoses were assessed with the Autism Diagnostic Interview-Revised (ADI-R) and Autism Diagnostic Observation Schedule (ADOS); rather, we relied on the DSM-5 criteria, CARS scores, and a clinical perspective. Finally, analyses showed that children who did not complete the PPVT-C were likely to be younger and had more severe autism than those who completed it. Although there was no significant difference in family income between the complete and incomplete groups, there was a trend in which ASD patients from lower-income families had more difficulty completing the test. As children with more severe autism or lower family SES have a higher risk of poor sleep (45), our results may not be generalizable to children with those risk factors. In addition, because children with ASD were excluded if they had taken any medication during the previous 6 months, we only studied Chinese preschool-aged children with ASD who were not taking medications. In China, behavioral treatment or/and cognitive-behavioral treatments are the first line of intervention to improve daytime function in preschoolers with ASD; if other treatments do not seem feasible or behavioral problems are particularly obvious, the cautious use of pharmacologic treatment is considered (54). Most children in the ASD group were classified as having low/moderate ASD based on CARS scores (34.54 ± 3.80), so none of them were taking medicine to treat daytime function, and only six children with ASD used supplemental melatonin to treat insomnia according to their medication history. Those children were excluded from the analysis. As such, caution is warranted regarding the generalization of the results on the relationship between sleep disturbances and daytime function.

## Conclusion

The current study assessed the culture-specific prevalence of sleep problems in preschool-aged children with ASD and age-matched TD children in China and revealed that sleep problems are common in this population. We also explored the subjects' sleep pattern characteristics in detail and concluded that interventions focused on sleep problems might improve emotional/behavior problems and repetitive behaviors in Chinese preschool-aged children with ASD. These findings emphasize the importance of screening and developing specific treatments and interventions. Further research should focus on promoting knowledge of sleep among pediatricians, relevant psychiatrists, and clinicians and establishing healthy sleep hygiene practices for preschool-aged children with ASD in China.

## Data Availability Statement

The datasets generated for this study will not be made publicly available because the data includes participant privacy. Requests to access the datasets should be directed to the corresponding author.

## Ethics Statement

The studies involving human participants were reviewed and approved by the ethics committee of Tianjin Medical University. Written informed consent to participate in this study was provided by the participants' legal guardian/next of kin.

## Author Contributions

Y-QK collected the data, conducted all data processing, and wrote the manuscript. X-RS collected some of the CSHQ data. G-FW assisted with manuscript review and revision. Y-YS trained the evaluator and conducted quality control assessments. P-YL collected some of the data from children with ASD. XZ assisted with the study design and funding and contributed to manuscript review and revision.

## Funding

This work was supported by grants from the National Nature Science Foundation of China (No. 81773459 and No. 81973070).

## Conflict of Interest

The authors declare that the research was conducted in the absence of any commercial or financial relationships that could be construed as a potential conflict of interest.
