# Supplementary material for: Sleep Problems Influence Emotional/Behavioral Symptoms and Repetitive Behavior in Preschool-Aged Children With Autism Spectrum Disorder in the Unique Social Context of China
Source: Front Psychiatry. 2020 Apr 16;11:273. doi: 10.3389/fpsyt.2020.00273 (PMC7179767; doi:10.3389/fpsyt.2020.00273)
Supplement: Supplementary file 1 [file Table_1.docx]

**Table S1 The characteristics of the children with ASD who whether or not completed the study.**

|  | ASD subgroup  n=114 | ASDs were ex-cluded subgroup  n=138 |  |  |
| --- | --- | --- | --- | --- |
|  | Mean±SD/ n(%) | Mean±SD/ n(%) | t*/χ*^2^ | *P* |
| Age(years) | 5.39±1.11 | 4.93±1.15 | 3.197 | 0.002 |
| **Gender** |  |  | 0.480 | 0.488 |
| Male | 94(82.5) | 109(79.0) |  |  |
| Female | 20(17.5) | 29(21.0) |  |  |
| Paternal age | 34.93±4.77 | 34.66±4.37 | 0.469 | 0.639 |
| Maternal age | 33.60±3.69 | 33.56±4.07 | 0.078 | 0.938 |
| **Paternal education level** |  |  | 0.001 | 0.971 |
| High school or below | 46(40.4) | 56(40.6)) |  |  |
| Undergraduate or above | 68(59.6) | 82(59.4) |  |  |
| **Maternal education level** |  |  | 0.431 | 0.512 |
| High school or below | 36(31.6) | 49(35.5) |  |  |
| Undergraduate or above | 78(68.4) | 89(64.5) |  |  |
| **Monthly family income** |  |  | 5.935 | 0.051 |
| <3000 RMB* | 9(7.9) | 21(15.2) |  |  |
| 3000-6000 RMB* | 37(32.5) | 54(39.1) |  |  |
| >6000 RMB* | 68(59.6) | 63(45.7) |  |  |
| CARS | 34.54±3.80 | 36.51±4.24 | -3.863 | 0.000 |

* 1 RMB=0.14 US Dollars
